# Maternal Psychotherapy Addressing Generational Stress is Associated with Lower Preterm Birth Risk: The Pforzheim Study

**DOI:** 10.1177/26884844251394236

**Published:** 2025-11-06

**Authors:** Gerlinde AS. Metz, Tanzi D. Hoover, Rupert Linder

**Affiliations:** ^1^ Canadian Centre for Behavioural Neuroscience, Department of Neuroscience, University of Lethbridge, Lethbridge, Canada.; ^2^ Southern Alberta Genome Sciences Centre, University of Lethbridge, Lethbridge, Canada.; ^3^ Private Practice for Gynecology, Psychosomatics and Psychotherapy, Birkenfeld, Germany.

**Keywords:** premature birth, stress, salutogenesis, intergenerational, transgenerational, psychosomatic, social support, cesarean section, newborn health, maternal health, mental health, birth weight, birth outcome

## Abstract

**Introduction:**

Maternal distress is a significant risk factor for adverse pregnancy and birth outcomes across generations. Psychotherapy can reduce distress and build resilience, potentially mitigating the effects of inter- and transgenerational stress.

**Methods:**

This study examined whether an integrated, personalized psychotherapy approach focused on inter- and transgenerational stress can be associated with improved pregnancy and birth outcomes. Records from 239 women and their babies were collected between 2004 and 2012 at a private clinic specializing in gynecology, obstetrics, psychosomatics, and psychotherapy in Germany. Pregnant women received routine gynecological care alongside complementary psychotherapeutic interventions, including system-oriented psychosomatic therapy, solution-focused therapy, salutogenesis, and couple therapy, with emphasis on inter- and transgenerational stress. These data were compared with birth records from regional standard care deliveries in the city of Pforzheim (*n* = 18,690) and the state of Baden-Württemberg (*n* = 815,832).

**Results:**

The findings showed that complementary psychotherapy was associated with lower rates of primary and secondary cesarean sections, fewer vaginal surgical procedures, a higher proportion of spontaneous births, and a lower preterm birth rate compared with standard care. Women receiving psychotherapy had an 18.2% higher likelihood of spontaneous birth and a lower observed preterm birth rate (2.2%) than those in standard care (9.0%). Infants of mothers in the psychotherapy group were heavier, taller, and had larger head circumferences.

**Conclusion:**

In this study, psychotherapeutic counseling within standard gynecological care was linked to longer pregnancy duration, improved birth mode, and greater birth weight. These findings suggest psychotherapy can be a safe, preventive approach during pregnancy to support maternal well-being and promote healthier outcomes for newborns.

## Introduction

Pregnancy is a period of heightened vulnerability for both mother and child. About 80% of pregnant women experience distress during pregnancy, which may increase the risk of preterm birth (PTB).^1^ At least 1 in 10 mothers suffers from antepartum depression or anxiety disorders with rates rising under distress and additional vulnerability after childbirth.^2–4^ Depression during pregnancy may double the PTB risk and lead to low birth weight, though the strength varies by measurement method and geography.^5,6^ At least 40%–45% of spontaneous PTB cases are arguably linked to adverse maternal environments, such as severe distress and low socioeconomic status.^7^

PTB results from multiple interacting physiological and pathological pathways that can prematurely trigger myometrial activation, cervical remodeling, and membrane rupture.^8^ Chronic stress, through hypothalamic–pituitary–adrenal (HPA) axis activation, may promote a proinflammatory state that disrupts pregnancy maintenance.^1,9^ Stress-induced HPA activation amplifies pathways leading to labor, including Toll-like receptors that mediate cytokine and chemokine production.^10,11^ Activation of interleukin-1β and interleukin-6 establishes an inflammatory microenvironment.^12,13^ Placentally synthesized corticotropin-releasing hormone (CRH) rises exponentially in pregnancy, influencing parturition timing *via* endocrine, autocrine, and paracrine effects.^14,15^ CRH stimulates nuclear factor-κB (NF-κB) activity, which promotes contraction-associated proteins and cytokines.^16^ NF-κB activation inhibits progesterone receptor B, causing functional progesterone withdrawal, while increased estrogen receptor activity and estriol levels enhance myometrial contractility and cervical ripening.^17,18^ These endocrine, immune, and structural changes converge to precipitate PTB.

The pathway to PTB can influence offspring development and long-term health, including poor fetal growth, delayed psychomotor and cognitive maturation, and dysregulated stress responses.^1,19^ During the COVID-19 pandemic, fear of infection was associated with a 192 g reduction in birth weight and a 6.1-day reduction in gestational age.^20^ Maternal depression and anxiety can impair interpersonal functioning,^21^ bonding, and child development.^22^ Biological effects of maternal stress may also increase PTB risk in future generations.^23,24^ These associations highlight the importance of improving maternal mental health to promote health for both mother and child.

Evidence-based interventions aim to reduce maternal gestational stress and improve pregnancy outcomes. Psychosomatic approaches in obstetrics and gynecology address pregnancy, childbirth, reproductive health, infertility, trauma, and mental health.^25–27^ Psychotherapeutic and psychosocial treatments can prevent prepartum depressive symptoms and stress.^28^ Even short-term interpersonal psychotherapy can improve maternal well-being and child outcomes,^28,29^ with greater benefits from more frequent counseling in a dose-response effect.^30^ Earlier findings and a recent randomized controlled trial showed that reducing maternal depression through psychotherapy is linked to longer gestation and higher chances of full-term births.^31,32^ Furthermore, inclusive relationship coaching, specifically for minority and low-income women, was also shown to improve birth outcomes.^33^ Psychological and social support may lower PTB rates in high-risk mothers.^34,35^

The present study evaluated an integrated psychotherapy approach, through combining personalized and system-oriented psychosomatic therapy, solution-focused therapy, salutogenesis, and couple therapy, on pregnancy and birth outcomes in a private gynecological practice near Pforzheim in the state of Baden-Württemberg, Germany. Pforzheim hosts one of Germany’s oldest Early Care Networks (*Netzwerk Frühe Hilfen*), providing multidisciplinary perinatal support.^27^ The city faces significant socioeconomic challenges, with high child poverty, unemployment, and 73% of youth having a migration background.^27,36^ Pforzheim also experienced severe World War II destruction, generating pervasive intergenerational trauma. The intervention focused on mitigating parental (including intergenerational) trauma and potential psychosomatic contributors to pregnancy complications.^26,29^ Data from 25 years of psychotherapeutic practice were compared with outcomes from standard care facilities in Pforzheim and Baden-Württemberg. A retrospective analysis suggests that psychotherapy addressing trauma and building resilience may be associated with more favorable pregnancy courses, childbirth outcomes, and newborn development.

## Methods

### Study design and population

The present study included data of 239 women treated with psychotherapy and their babies, for whom complete birth records were available. Data were collected between 2004 and 2012 by a private gynecology–psychotherapy–psychosomatics office in Birkenfeld, Germany. The nearby city of Pforzheim with a population of 125,000 is located in the state of Baden-Württemberg, southwest Germany, near the northern edge of the Black Forest. The psychotherapy data were compared with in standard care data based on anonymized electronic medical records of mother–child dyads from the area of Pforzheim (*n* = 18,690) and the state of Baden-Württemberg (*n* = 815,832) using the online data collection agency Geschäftsstelle Qualitätssicherung im Krankenhaus (GeQiK). GeQiK is a semi-public office, which collects data for quality assurance among hospitals in the Baden-Württemberg region. GeQiK records all of the births that have occurred in a hospital, including newborn outcomes, medications, examination of umbilical cord blood, emergency cesarean sections, and premature births.

Pregnant women in the psychotherapy program were provided with standard gynecological medical treatments combined with psychotherapeutic interventions during scheduled clinic visits during the length of their pregnancy. Visits typically lasted 30–50 min, with approximately 20 min of gynecological examination and treatment and the remainder devoted to counseling, during each clinic visit. On average, 16 visits at the private office were provided during pregnancy, compared with an average of 12 visits in the Pforzheim and Baden-Württemberg standard care comparison groups. Personalized counseling and psychodynamic therapy included personalized system-oriented psychosomatic therapy, solution-focused therapy, salutogenesis, and couple therapy interventions. Themes around partner relationships, work and occupational stress, and past personal trauma were often targeted in treatment, including focus on maternal support systems and enhancing coping skills. A special focus was placed on parental (intergenerational) trauma and potential generational conflicts, such as transgenerational trauma, in order to assess the origins of current pregnancy complications.^26,29^

GeQiK had reviewed and approved the use of their data for this study. This study was approved by the Landesärztekammer Baden-Württemberg (#F-2018-093) and the University of Lethbridge Human Participant Research Committee (#2020-062).

### Control group identification and inclusion criteria

Psychotherapy participants with complete newborn outcome data (*n* = 239) were compared with women who received standard obstetrical care in the same region. Standard-of-care controls were identified retrospectively from birth records maintained by obstetric facilities in Pforzheim (*n* = 18,690) and from the official perinatal registry of the state of Baden-Württemberg (*n* = 815,832). Controls were selected from deliveries occurring during the same period as the psychotherapy cases (2004–2012) to ensure temporal comparability. Inclusion criteria for controls were: singleton pregnancy, live birth, and documented maternal and newborn data for birth mode, gestational age, and anthropometric outcomes. Where available, maternal age, parity, and obstetric risk factors were reviewed to confirm similarity with the psychotherapy group. Deliveries involving elective transfers from outside the region, incomplete records, or congenital anomalies were excluded. For each psychotherapy participant, control data were drawn from the aggregate standard-care dataset. No participants were excluded because of the absence of suitable controls, as the regional datasets encompassed all births meeting the inclusion criteria during the study period.

### Mode of delivery

The mode of delivery was identified *via* G-DRG (German Diagnosis-Related Groups System). G-DRGs are used in billing and contain information on individual diagnoses and procedures performed during their clinic and hospital visit. The G-DRG System categorizes births into vaginal birth and cesarean section. Only births in a hospital were included. Birth weight of 2,500 g and lower was defined as low birth weight, and birth weight above 4,500 g was defined as fetal macrosomia.

### Statistical analyses

Data obtained from pregnant women who received care in the private gynecology office between 2004 and 2012 were compared with GeQiK records of Pforzheim and Baden-Württemberg between 2004 and 2012. All data analyses were performed using SAS version 9.3 (SAS Institute, Cary, NC, USA). Comparisons between groups were made using chi-square or *t-*tests, respectively, depending on the data type. Density plots were used for real-valued and bar plots for categorical data. Each chart compares the births of women in corresponding locations of either Pforzheim or Baden-Württemberg. Categorical variables are represented with crosstabs and bar charts. Numerical values are presented as mean ± standard deviation. *p* Values of less than 5% were considered as statistically significant.

## Results

### Complementary psychotherapy is associated with improved pregnancy and birth outcomes

**FIG. 1.**
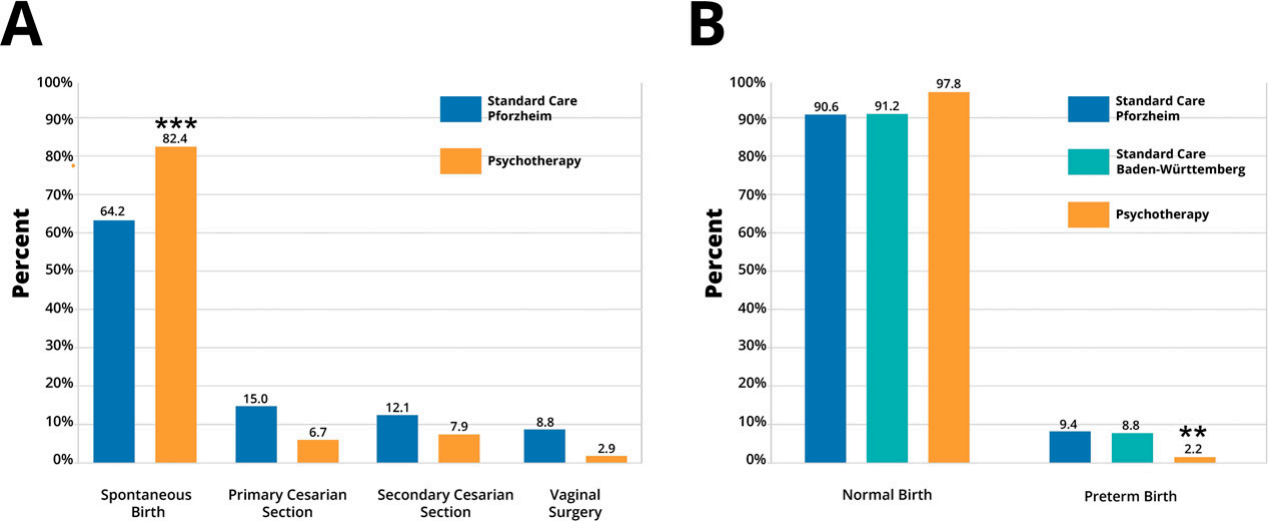
Prenatal psychotherapy was associated with more favorable pregnancy outcomes. **(A)** Comparison of mode of delivery in the psychotherapy group compared with the general population receiving standard care in Pforzheim and Baden-Württemberg (GeQiK data). Complementary psychotherapy in gynecological practice was associated with a higher incidence of spontaneous births due to lower risk of primary and secondary cesarean section and vaginal surgery. **(B)** Psychotherapy in addition to standard gynecological care was linked to higher chances of spontaneous births and reduced risk of preterm birth in comparison with standard care in both Pforzheim and Baden-Württemberg (GeQiK data). **p < 0.01, ***p < 0.001.

The demographics of mothers included in this study revealed an average age of 29.97 years at the time of birth in the city of Pforzheim, 30.64 years in the state of Baden-Württemberg, and 31.89 years in the Psychotherapy group, also located in Pforzheim (Table 1). The addition of psychotherapy treatment to standard gynecological care was associated with significantly improved pregnancy and birth outcomes. In particular, the complementary use of psychotherapy in gynecological practice was associated with a significantly higher rate of spontaneous births (82.4%) compared with the rate of spontaneous births among mothers who received standard care without psychotherapy in Pforzheim (64.2%) (*p* < 0.0001; Fig. 1A). This difference was linked to an overall reduction in primary and secondary cesarean sections, and reduction in vaginal surgical procedures among mothers who received psychotherapy compared with the general population receiving standard care without psychotherapy. Primary cesarean sections in the psychotherapy group were performed 6.7% of the time, compared with 15% in both Pforzheim and the state of Baden-Württemberg. Secondary cesarean sections in the psychotherapy group were performed 7.9% of the time, compared with 12.1% in Pforzheim and 14.9% in the state of Baden-Württemberg. Vaginal surgical procedures in the psychotherapy group were performed 2.9% of the time compared with 8.8% in both Pforzheim and Baden-Württemberg. Thus, complementary psychotherapy treatment reduced the rate of primary cesarean section by 45%, secondary cesarean section by 35%, and the rate of vaginal surgery by 67%.

**Table 1.** Summary of Maternal Age (Years) in the Psychotherapy Cohort Compared with Pforzheim and the State of Baden-Württemberg

	Mean	SD	Min	Max	*n*	Difference	*t*-statistic	*p* value
Pforzheim	29.97	5.48	14	51	18,690	−1,93	−5.37	<0.001
Psychotherapy	31.89	5.50	17	45	239			
Baden-Württemberg	30.64	5.36	10	59	81,5832	−1,25	−3.51	<0.001
Psychotherapy	31.89	5.50	17	45	239			

**FIG. 2.**
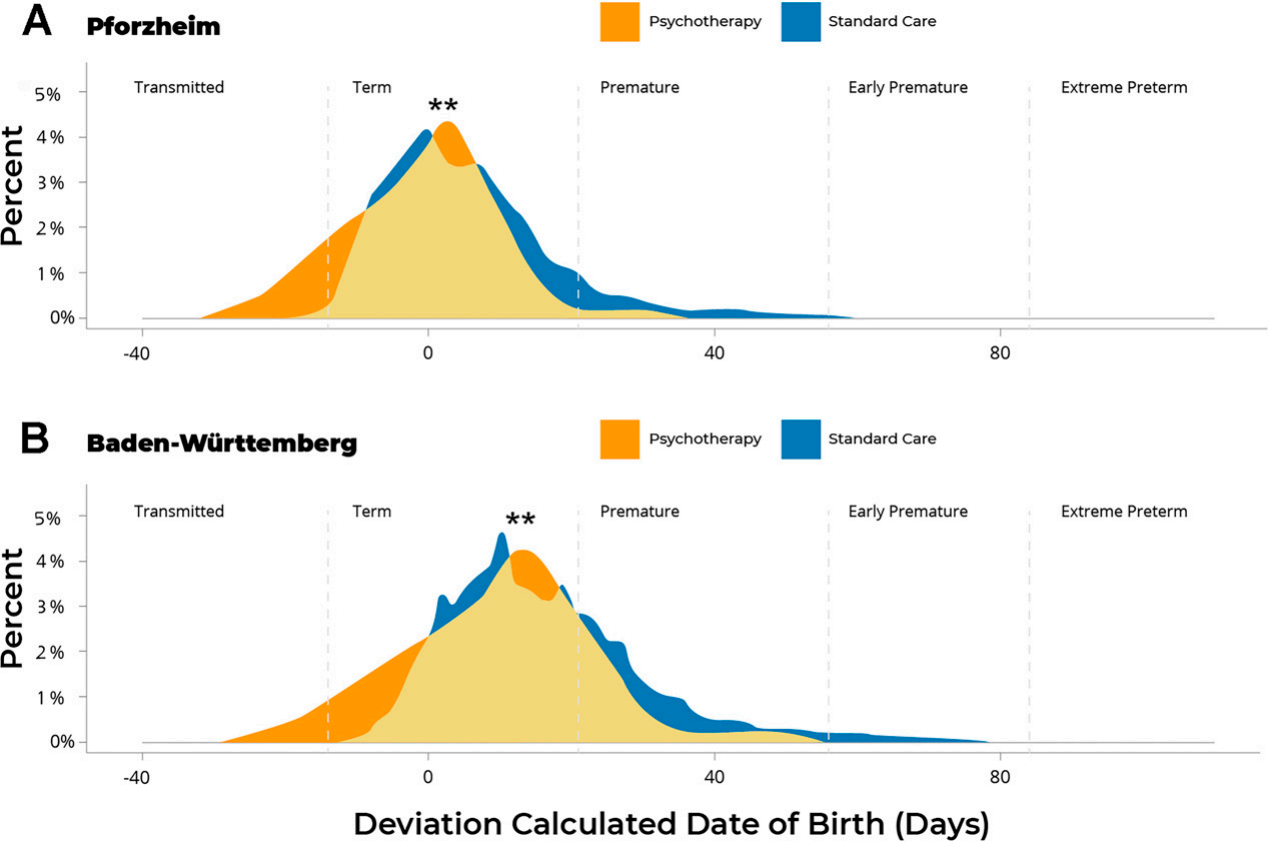
Prenatal psychotherapy was associated with a shift in gestational duration towards term delivery. Deviation of gestational duration from the calculated due date in the psychotherapy group compared with the general population receiving standard care in **(A)** Pforzheim and **(B)** Baden-Württemberg (GeQiK data). Compared with the psychotherapy group, newborns in standard care were delivered on average 6.6 days earlier in Pforzheim and 6.15 days earlier in Baden-Württemberg. Thus, psychotherapy was associated with a significantly higher prevalence of term births compared with the general population in both Pforzheim and Baden-Württemberg. **p < 0.01.

**FIG. 3.**
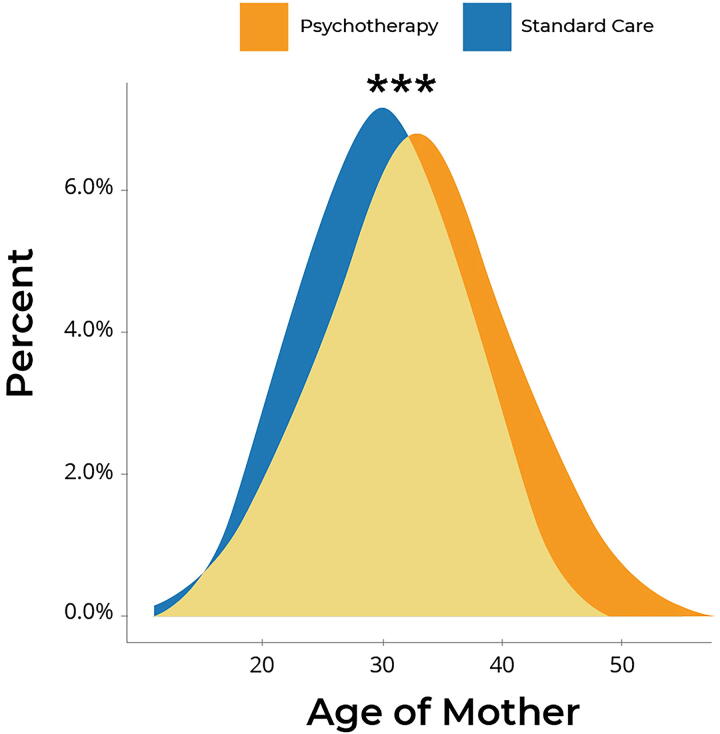
Maternal age at time of birth. The age profile among mothers treated in the Standard Care group (mean 30.3 years) was younger compared with mothers treated in the Psychotherapy group (mean 31.89 years). ***p < 0.001.

In addition to mode of delivery, inclusion of psychotherapy in standard gynecology practice affected gestational length. Psychotherapy was associated with a lower rate of PTB (<37 weeks gestation) when compared with standard care without psychotherapy. The rate of PTB in the psychotherapy group was reduced to 2.2% compared with 9.4% in Pforzheim and 8.8% in Baden-Württemberg (*p* < 0.001; Fig. 1B). Overall, psychotherapy was associated with approximately 3%–4% higher chance of term birth compared with both Pforzheim and Baden-Württemberg, and 3% lower risk of late births. As shown in Figure 2, complementary psychotherapy reduced the overall deviation in gestational length from term births and significantly increased the prevalence of term births compared with standard care without psychotherapy in both Pforzheim and Baden-Württemberg (*p* < 0.01; Fig. 2A and B, respectively). Thus, mothers with standard care who did not receive psychotherapy delivered their newborns 6.6 days earlier in Pforzheim and 6.15 days earlier in Baden-Württemberg compared with mothers who received complementary psychotherapy (Table 2). The distributions illustrate that births in the psychotherapy group were more likely to be full-term or late. In the psychotherapy group, 97.8% of births occurred at term compared with 90.6% in Pforzheim and 91.2% in Baden-Württemberg (Fig. 2A and B, respectively). Given that advanced maternal age is a critical risk factor for PTB, the shift in gestational length in the psychotherapy group was remarkable given that mothers receiving complementary psychotherapy were on average 1.9 years older than mothers receiving standard care in Pforzheim (*p* < 0.001; Fig. 3) and 1.3 years older than mothers receiving standard care in Baden-Württemberg (*p* < 0.001). Thus, complementary psychotherapy ameliorated the elevated risk of PTB in older mothers.

**Table 2.** Summary of Gestational Duration in Term Births (Days from the Expected Due Date, *i.e.,* Gestational Age Deviation) Based on Date of Birth in the Psychotherapy Cohort Compared with Pforzheim and the State of Baden-Württemberg

	Mean	SD	Min	Max	*n*	Difference	*t*-statistic	*p* value
Pforzheim	6.28	15.00	−25	155	18,690	6.60	7.33	<0.01
Psychotherapy	−0.31	13.54	−28	131	239			
Baden-Württemberg	5.84	14.84	−30	273	815,832	6.15	6.89	<0.01
Psychotherapy	−0.31	13.54	−28	131	239			

### Complementary psychotherapy is associated with improved newborn outcomes

**FIG. 4.**
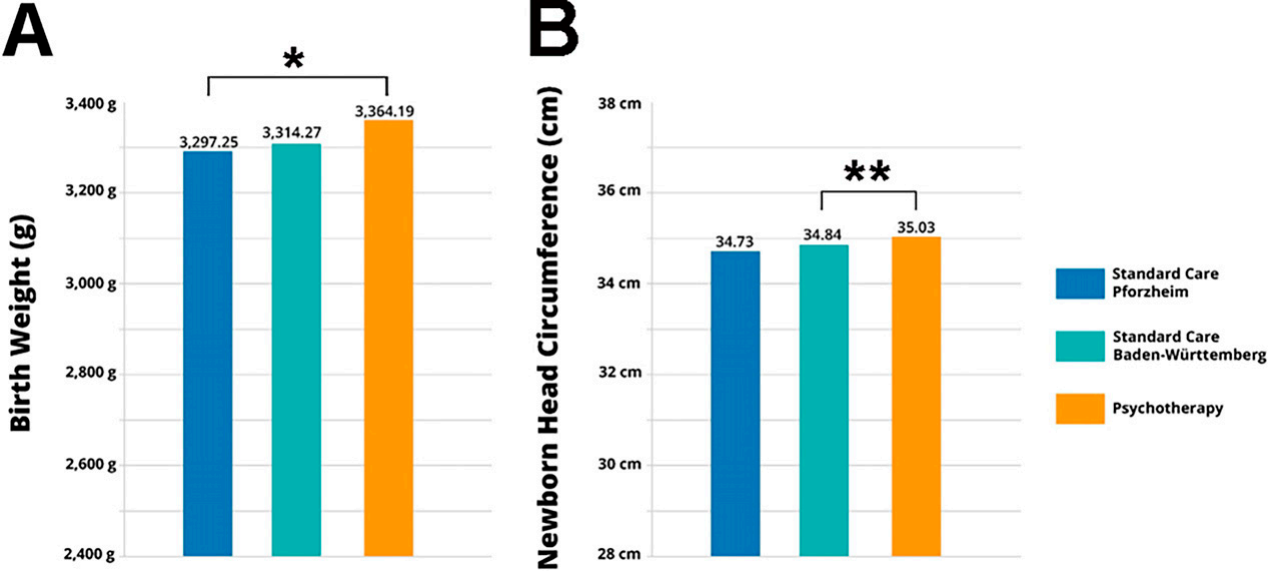
Prenatal psychotherapy improved newborn health outcomes. **(A)** Birth weights were higher in the psychotherapy group when compared with the general population receiving standard care in Pforzheim. **(B)** Newborns born to mothers who received psychotherapy showed larger head circumferences compared with the general population receiving standard care in Baden-Württemberg. *p < 0.05, **p < 0.01.

Newborn statistics also revealed significant effects of maternal prenatal psychotherapy. Newborns born to mothers who received psychotherapy (*n* = 239) were on average 67 g heavier (3,364.2 +/− 480.2 g) when compared with the general population receiving standard care in Pforzheim (3,297.3 +/− 576.3 g; *p* < 0.05; Fig. 4A). Birth weights in the psychotherapy group were not statistically different from Baden-Württemberg (*p* < 0.11; Table 3). Mothers who received prenatal psychotherapy overall gave birth to fewer newborns below 2,500 g birth weight. Newborns born to mothers who received psychotherapy were also significantly taller (51.8 +/− 2.7 cm) compared with the general population receiving standard care in Pforzheim (51.36 +/− 2.9 cm; *p* < 0.01) and Baden-Württemberg (51.1 +/− 3.0 cm; *p* < 0.001; Table 4). In addition, newborns born to mothers who received psychotherapy showed larger head circumference of 35.0 cm compared with 34.8 cm in Pforzheim (n.s.) and 34.7 cm in Baden-Württemberg (*p* < 0.01; Table 5 and Fig. 4B).

**Table 3.** Summary of Birth Weight (g) in the Psychotherapy Cohort Compared with Pforzheim and the State of Baden-Württemberg

	Mean	SD	Min	Max	*n*	Difference	*t*-statistic	*p* value
Pforzheim	3,297.25	576.28	500	5,620	18,690	−66.95	−2.12	<0.05
Psychotherapy	3,364.19	480.17	1,530	4,580	239			
Baden-Württemberg	3,314.27	576.21	500	9,705	18,690	−49.92	−1.60	0.11
Psychotherapy	3,364.19	480.17	1,530	4,580	239			

**Table 4.** Summary of Newborn Height (cm) in the Psychotherapy Cohort Compared with Pforzheim and the State of Baden-Württemberg

	Mean	SD	Min		Max	*n*	Difference	*t*-statistic	*p* value	
Pforzheim	51.13	3.02	0	99		815,832	−0.71	−4.03	<0.001	
Psychotherapy	51.84	2.68	42	59		239				
Baden-Württemberg	51.36	2.90	10	62		18,690	−0.48	−2.72	<0.01	
Psychotherapy	51.84	2.68	42	59		239				

**Table 5.** Summary of Newborn Head Circumference (cm) in the Psychotherapy Cohort Compared with Pforzheim and the State of Baden-Württemberg

	Mean	SD	Min	Max	*n*	Difference	*t*-statistic	*p* value
Pforzheim	34.73	1.76	0	99	815,832	−0.30	−2.82	<0.01
Psychotherapy	35.03	1.60	28.5	39	239			
Baden-Württemberg	34.84	1.58	18	52	18,690	−0.19	−1.74	0.08
Psychotherapy	35.03	1.60	28.5	39	239			

## Discussion

The present study showed that complementary antepartum psychotherapeutic intervention in standard gynecological practice, aimed at addressing lifetime, inter- and transgenerational stresses, was associated with lower rates of cesarean section, vaginal surgical procedures and PTB, and higher birth weight. These associations are particularly notable considering that the psychotherapy treatment group included women of generally older maternal age, a demographic typically at higher PTB risk. Improvements in pregnancy and birth outcomes were observed relative to the general local population giving birth in hospitals in the Pforzheim and Baden-Württemberg regions. The findings align with the view that psychobehavioral risk and inter- and transgenerational stress may be important determinants of adverse pregnancy and birth outcomes.^1,23,24,31^ Overall, the results suggest that psychotherapy supports psychosomatic health across all stages of birth.

The multiprofessional initiatives in Pforzheim have collected health data since the early 1990s, with activities expanding significantly in 2005 through the formation of the Early Care Network. This network implements collaborative prevention strategies delivered by physicians, midwives, nurses, psychologists, social workers, and other professionals.^27^ For example, physicians and midwives cooperate in joint intake evaluations and birth planning, promote salutophysiology, and participate in regular interdisciplinary team meetings and conferences. Premature births and birth complications are systematically reviewed by pediatricians, neonatologists, obstetricians, midwives, and pediatric surgeons. This highly collaborative community framework, designed to strengthen long-term maternal and child health trajectories, fosters an environment that is especially supportive for young families, pregnant women, and newborns.^27,37^ The present data indicate that adding psychotherapy to this robust maternal care model further promotes healthy pregnancy outcomes.

Antenatal depression represents a significant risk factor in adverse pregnancy and birth outcomes, such as increased risks of preeclampsia, PTB, low birth weight, small-for-gestational-age infants, and other complications.^1,6^ In a systematic review, Accortt et al. reported a significant association between antenatal depression and shorter gestation in 25% of studies, whereas over half of studies reported an association between antenatal depression and low birth weight.^38^ In addition, maternal lifetime stress, antepartum depression, and anxiety are significant risk factors for inflammatory processes that facilitate outcomes such as PTB.^39^ Moreover, a maternal history of abuse prior to pregnancy has been strongly linked to increased risk of PTB and low birth weight.^40^ Maternal stress was reported more frequently among women who delivered preterm (54%) compared with the overall sample (23%) in one study.^41^ Mechanistically, these associations are mediated by dysregulation of the HPA axis, chronic inflammation, altered placental function, and behavioral factors linked to cumulative lifetime and ancestral stresses.^1,42^ Because antenatal depression and adverse birth outcomes show clear disparities across populations,^43^ it is critical to develop targeted interventions for the most vulnerable mothers to mitigate biological stress pathways and promote maternal resilience.

In the present study, consultation and counseling incorporated biographical, psychological, social, sociodemographic, biological, and somatic information.^26^ Particular emphasis was placed on the biographical inter- and transgenerational history of birth complications when evaluating risk and designing the therapeutic approach.^26,29^ Experimental studies underscore the relevance of this strategy, demonstrating that cumulative lifetime stress, prenatal and intergenerational trauma,^1,44^ and the stress of even remote ancestors can increase PTB risk and negatively affect child health trajectories.^24^ Evidence suggests that reducing the impact of stress during pregnancy through psychotherapy and counseling can improve maternal and neonatal health.^34,45–47^ For example, an integrated therapeutic approach aimed at reducing psycho-behavioral risks in African–American women significantly lowered the incidence of very PTB compared with standard care.^31^ The present findings emphasize the benefits of integrated psychosomatic and psychotherapeutic interventions in routine prenatal care to promote healthy pregnancies.

It has been shown that participation in psychotherapeutic and psychosomatic interventions during pregnancy can reduce perceived stress, anxiety, postpartum depression, and intimate partner violence, while also promoting maternal mental health, positive pregnancy and birth outcomes.^34,46–48^ These approaches may translate into improved pregnancy outcomes, with potential long-term benefits for postpartum recovery and newborn development. For example, higher positive affect during pregnancy was linked to longer gestation and reduced PTB risk, with individual improvement correlating with gestational length.^49^ Notably, better maternal mood in the second trimester predicted outcomes independently of perceived stress.^49^ A central mechanism underlying the efficacy of these interventions may be their capacity to build stress resilience and attenuate stress-associated health outcomes, including depression, anxiety, and pro-inflammatory states that can precipitate adverse pregnancy events. Moreover, social support is arguably integral to these benefits. Supportive social environments improve birth weight, particularly in ethnic minorities and can buffer psychosocial stress by strengthening coping resources.^9,39^ Prenatal programs with personal interactions enhance birth weight and reduce PTB, cesarean section, and other risks,^50,51^ with psychosocial care lowering low birth weight risk by 40%.^52^ Experimental evidence also confirmed that social support fosters resilience and mental health and reduces PTB risk linked to social isolation.^44,53–55^ Psychotherapy interventions may act through physiological, metabolic, and epigenetic pathways, as shown experimentally.^56^ Such biological processes may aid in promoting maternal recovery, mother–infant bonding, child development, and healthier pregnancies in subsequent generations.^1^

The present psychotherapeutic approach recognized that pregnancy care must balance physical and emotional wellbeing.^26,57,58^ Transgenerational adverse experiences can significantly influence premature birth risk,^29^ with early emotional constellations, such as the mother’s reaction to an intended or unintended pregnancy, shaping the mother–child dyad.^58^ Maternal responses to pregnancy may also be shaped by unconscious imprinting from previous generations, including parental experiences.^58^ Consequently, individual biographical details and family history of birth complications were central to the therapeutic plan. Past events such as premature rupture of membranes, hospitalization, or chorioamnionitis can impose substantial psychological stress on mothers and newborns.^59^ Notably, standard treatment clinics may have received more low-risk pregnancies, whereas higher-risk mothers in the region were more likely referred to the Linder practice, further emphasizing the therapy’s benefits among this higher-risk group. Addressing psychological and practical barriers to treatment is critical, and therapeutic benefits increase when family members are engaged.^60^ For example, the Family Foundations program, which promotes coparenting quality, significantly buffered the adverse effects of excessive maternal cortisol on birth weight, gestational length, and infant hospital days.^50^ These results underscore that strong social relationships reduce adverse pregnancy outcomes, especially among higher-risk women.

It should also be noted that stress and depression during pregnancy can increase the likelihood of smoking, substance use, and reduce the probability that pregnant women seek prenatal care,^61^ thereby perpetuating the cycle of stress and adverse pregnancy outcomes. Women experiencing high stress relative to personal capital, defined as multidimensional resources that aid in coping during stress exposure, were more likely to have lower gestational age and to experience at least one pregnancy complication, including PTB.^19^ Risk factors for depression in pregnancy include a family history of depression, childhood maltreatment, single motherhood, cigarette smoking, low income, domestic violence, and insufficient social and partner support.^26,37,40^ Pharmacological treatment with antidepressants may not be advisable during pregnancy, as it has been associated with increased risks of PTB, spontaneous abortion, neurological and cardiovascular abnormalities, and lower gestational age.^62^ Ideally, treatment of depression in pregnant women should focus on enhancing coping skills and resources, which can be achieved through psychotherapeutic interventions and other psychobehavioral strategies. The efficacy of the intervention in the present study supports the value of non-pharmacological approaches for managing mood disorders during pregnancy.

## Limitations

While the present findings are consistent with prior studies reporting benefits of psychotherapeutic interventions for reducing adverse pregnancy outcomes, several limitations should be acknowledged. It is possible that pre-existing differences between women who received psychotherapy compared with those who received standard care contributed to the observed findings. Such differences may include sociodemographic characteristics, psychological health status, prior birth experiences, perceived need for care, or attitudes toward mental health interventions. The effect size might also have been affected by the fact that women referred to the psychotherapy program were potentially at higher risk. Moreover, women who actively sought therapy may differ psychologically from those who did not in ways that could influence pregnancy outcomes, such as higher health literacy, greater motivation to engage with health care providers, or stronger existing social support networks. This raises the possibility of selection bias, whereby observed outcomes may in part reflect underlying participant characteristics rather than the effects of psychotherapy itself. Because participation in therapy was not randomly assigned, causal inferences cannot be drawn. Future research should therefore incorporate randomization, comprehensive baseline data collection, and adjustment for potential confounding factors.

Although the present study was part of a long-term project with an overall larger cohort, which ran from 1987 to 2012, the lack of a matched control group limits interpretation of the results. Data collection was restricted to a single gynecology–psychotherapy–psychosomatics office and was based on a regionally specific population. Findings may therefore not necessarily be generalizable to other socio-economic contexts or health care systems. However, the comprehensive GeQiK dataset used for comparison included all hospital deliveries in Pforzheim and Baden-Württemberg, thereby encompassing the specific mothers who underwent psychotherapy. It should be noted that the GeQiK dataset was limited to hospital deliveries, and the inclusion of home deliveries may have influenced the results.

## Conclusions

The present data suggest that psychotherapy and counseling may provide effective complementary treatments in support of a healthy pregnancy and delivery, as indicated by a significant reduction in primary and secondary cesarean sections, vaginal surgeries, PTB, and adverse newborn outcomes. These favorable outcomes were observed in spite of a higher proportion of high-risk mothers who participated in the psychotherapy program. The present comprehensive intervention involved a holistic approach to personalized psychotherapy, including aspects of physical psychotherapy, salutogenesis, couple therapy, and system-oriented psychosomatic therapy with emphasis on inter- and transgenerational trauma. The present findings therefore confirm the notion that lifetime and transgenerational psychobehavioral risk factors are associated with adverse pregnancy and birth outcomes. In spite of the study’s limitations and lack of mechanistic data, the results suggest that offering gynecological and obstetrical medical treatments combined with psychotherapy may promote maternal resilience and positively influence the physiological course of pregnancy and childbirth. Thus, complementary psychotherapy can be safely recommended throughout pregnancy to promote maternal and child health.

## Data Availability

Data from GeQiK and private practice are available upon request to the authors.

## Abbreviations Used

CRH: corticotropin-releasing hormone
G-DRG: German Diagnosis-Related Groups System
GeQik: Geschäftsstelle Qualitätssicherung im Krankenhaus
HPA: hypothalamic-pituitary-adrenal
NF-κB: nuclear factor-κB
PTB: preterm birth
TLR: Toll-like receptor
